# Mutation of microphthalmia-associated transcription factor (mitf) in zebrafish sensitizes for glomerulopathy

**DOI:** 10.1242/bio.040253

**Published:** 2019-02-04

**Authors:** Janina Müller-Deile, Heiko Schenk, Philipp Niggemann, Patricia Bolaños-Palmieri, Beina Teng, Alysha Higgs, Lynne Staggs, Hermann Haller, Patricia Schroder, Mario Schiffer

**Affiliations:** 1 Department of Nephrology, Hannover Medical School, Hannover 30625, Germany; 2 Department of Nephrology and Hypertension, University of Erlangen-Nurnberg, Erlangen 91054, Germany; 3 Division of Nephrology, Mount Desert Island Biological Laboratory, Salisbury Cove, ME 04609, USA

**Keywords:** MITF, Podocyte, Zebrafish, PAN, Glomerulopathy

## Abstract

Different glomerular diseases that affect podocyte homeostasis can clinically present as nephrotic syndrome with massive proteinuria, hypoalbuminemia, hyperlipidemia and edema. Up to now, no drugs that specifically target the actin cytoskeleton of podocytes are on the market and model systems for library screenings to develop anti-proteinuric drugs are of high interest. We developed a standardized proteinuria model in zebrafish using puromycin aminonucleoside (PAN) via treatment in the fish water to allow for further drug testing to develop anti-proteinuric drugs for the treatment of glomerular diseases. We noticed that fish that carry the nacre-mutation show a significantly higher susceptibility for the disruption of the glomerular filtration barrier following PAN treatment, which results in a more pronounced proteinuria phenotype. Nacre zebrafish inherit a mutation yielding a truncated version of microphthalmia-associated transcription factor/melanogenesis associated transcription factor (mitf). We hypothesized that the nacre mutation may lead to reduced formin expression and defects in cytoskeletal rearrangement. Based on the observations in zebrafish, we carried out a PAN treatment on cultured human podocytes after knockdown with MITF siRNA causing a rearrangement of the actin cytoskeleton.

## Abstract (precis)

**Summary:** We discovered that the mitf mutation, causative for nacre-albinism mutation in zebrafish, sensitizes for glomerulopathy-induced by puromycin aminonucleoside (PAN) and developed a fish water treatment-model for anti-proteinuric drug screenings.

## INTRODUCTION

Glomerular diseases that primarily affect podocyte function are often associated with the development of nephrotic syndrome which presents itself with proteinuria, hypoalbuminemia, hyperlipidemia and edema (Orth and Ritz, 1998). Focal segmental glomerulosclerosis (FSGS), membranous glomerulonephropathy (MGN), minimal change disease (MCD) and late stage diabetic nephropathy are most commonly associated with nephrotic syndrome. Thus far, therapeutic options for nephrotic syndrome are limited to supportive treatment options with RAAS-blockade, statins and treatment of other renal risk factors. In the case of FSGS, MCD and MGN immunosuppressive therapy is frequently added with a high rate of potentially life threatening side effects. Therefore, there is a high demand for novel and more podocyte-specific treatment concepts.

Puromycin aminonucleoside (PAN)-induced nephrosis in rats serves as a commonly used rodent model for human nephrotic syndrome. PAN specifically injures podocytes, leading to foot process effacement, actin cytoskeleton disorganization, as well as decreased expression and abnormal distribution of slit diaphragm proteins (Guan et al., 2004). In lower concentrations, PAN causes a mild and potentially reversible glomerular damage similar to minimal change disease, whereas at higher concentrations PAN treatment resembles components of human FSGS. Furthermore, in rats, injection of PAN decreases filtration rate and glomerular volume, and increases albumin excretion and podocyte foot process width in a dose-dependent manner (Löwenborg et al., 2000). Thus the PAN model is suitable to test for protective drugs to treat various glomerulonephropathies. Triptolide, for example, protects podocytes from PAN-induced injury *in vivo* and *in vitro* (Zheng et al., 2008). However, the use of rodent models for high-throughput drug screenings dose response and toxicity analysis is costly, labor intensive and requires large amounts of animals. As an alternative, the zebrafish larva is an ideal model to study glomerular diseases with many advantages compared to rodent models. The zebrafish pronephros consists of two pronephric ducts which are linked to a glomerulus, located at the midline with high similarity to the mammalian nephron (Drummond and Davidson, 2016). Moreover, the human and zebrafish genomes share a high degree of homology with around 70% of orthologous genes (Howe et al., 2013). Zebrafish have a high fecundity, produce a large number of offspring and the translucent larvae can be rapidly analyzed for a phenotype of interest. Furthermore, gene expression in zebrafish larvae can easily be influenced by specific knockdown or overexpression techniques such as morpholino injection, mRNA injection, microRNA injection, transcription activator-like effector nucleases (TALENs), zinc finger nucleases and CRISPR-Cas9 system (Hanke et al., 2013; Schenk et al., 2017; Müller-Deile et al., 2017).

Recently, PAN was also used in a zebrafish model for induction of acute podocyte injury (Rider et al., 2018). In this study, injection of PAN in the cardinal vein of zebrafish larvae at 3 days post-fertilization resulted in a loss of 70 kDa dextran and induced podocyte effacement. This technique works well but requires anaesthesia of the fish larvae and advanced microinjection skills to inject correctly and reproducible into the cardinal vein plexus of the 3 mm larva.

Because of the added complications of cardinal vein injections, we aimed to develop a less invasive method that produces a standardized glomerular proteinuria phenotype in zebrafish through PAN treatment via the fish water. This additionally facilitates the experiment making it possible to treat a higher number of fish without the added variability of additional manipulation. In previous experiments, we noted an inconsistent phenotype development with zebrafish from different genetic backgrounds by adding PAN to the fish water. We discovered that we had to optimize the treatment conditions and timing to develop a very consistent glomerular injury model. We identified differences in PAN sensitivity depending on the zebrafish strain background and we can give a possible molecular explanation for this observation.

We found that using our model in nacre (*nac^w2^*) zebrafish resulted in the most consistent phenotype. Nacre (*nac^w2^*) is a mutation in a zebrafish gene encoding a basic helix-loop-helix/leucine zipper transcription factor related to microphthalmia (mitf) (Lister et al., 1999). Previously, mitf was identified as a regulator of actin polymerization via formins (Carreira et al., 2006). Inverted formin 2, encoded by the INF2 gene, is a member of the formin family. In podocytes, INF2 plays a key role as a regulator of function and structure of the actin cytoskeleton (Safarikova et al., 2018) and mutations in INF2 are known to cause genetic forms of FSGS, which underlines the potential relevance of the mitf-INF2 axis for patients suffering from FSGS (Safarikova et al., 2018; Caridi et al., 2014).

In summary, this model can serve on the one hand as a tool to test drugs which are potentially beneficial for the integrity of the glomerular filtration barrier. On the other hand, the model could also be used to uncover mutations that cause a higher susceptibility to glomerular damage like the mitf mutation, but are not sufficient to affect glomerular function on their own.

## RESULTS

### Influence of genetic background on the proteinuria phenotype

**Fig. 1.**
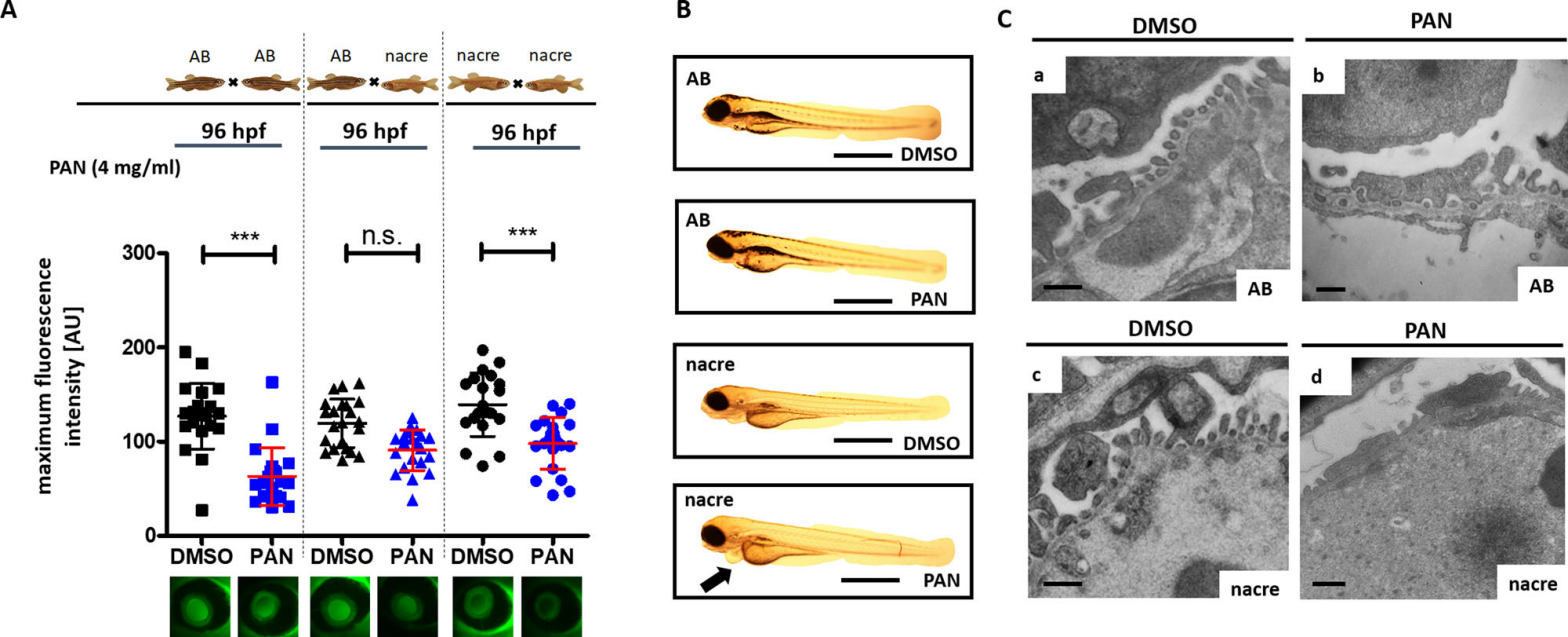
**Influence of genetic background on the proteinuria phenotype.** (A) Tg(*l-fabp*:eGFP-DBP) zebrafish backcrossed on homozygous for either the AB (AB fish) or the nacre (nac^w2^) background (nacre fish) as well as heterozygous zebrafish for nacre and AB background were treated with PAN at 46 h post-fertilization (hpf). Graph depicts max. fluorescence of circulating eGFP-DBP in the retinal vessel plexus of the fish eye 96 hpf. (B) Representative images of phenotypes of Tg*(l-fabp:*eGFP-DBP) zebrafish backcrossed on homozygous for either the AB (AB fish) or the nacre (nac^w2^) background (nacre fish) were treated with PAN (4 mg/ml) or DMSO at 46 hpf as indicated. Pictures were taken at 96 hpf. (C) Representative transmission electron microscopy images of the glomerular filtration barrier of Tg(*l-fabp:*eGFP-DBP) zebrafish backcrossed on homozygous for either the AB or the nacre (nac^w2^) background were treated with PAN (4 mg/ml) or DMSO at 46 hpf as indicated. Fish were collected at 120 hpf. Podocyte effacement after PAN treatment was more severe in nacre fish compared to AB fish. ***P<0.001, n.s., non-significant. Scale bars: (B) 500 μm, (C) 500 nm.

We wanted to test whether the genetic background of the zebrafish strains affects sensitivity towards PAN treatment. We used Tg(*l-fabp*:eGFP-DBP) zebrafish embryos from an intercross of a transgenic line in AB (AB×AB), nacre (*nac^w2^×nac^w2^*) or mixed (AB×*nac^w2^*) background for this experiment. All zebrafish larvae were treated with 4 mg/ml PAN at 46 hpf and fluorescent signal in the retinal vessels was detected at 96 hpf. Interestingly, zebrafish larvae from an intercross on nacre background were highly susceptible to PAN treatment resulting in proteinuria while zebrafish of AB background did not develop proteinuria (Fig. 1A). Consistent with the higher susceptibility towards proteinuria, nacre fish developed an edematous phenotype after PAN treatment (Fig. 1B). Of note is that the edema phenotype in this model system is very mild with discrete pericardial effusion compared to the severe edema that we frequently observe after genetic alterations (Müller-Deile et al., 2017, 2016b, 2018; Teng et al., 2016; Ashworth et al., 2010).

To assess if the loss of plasma proteins after PAN treatment was due to damage on the glomerular filtration barrier, we performed electron microscopy analysis of the zebrafish at 120 hpf. Podocytes, glomerular endothelial cells and glomerular basement membrane looked healthy in AB as well as nacre zebrafish lines (Fig. 1Ca,c). PAN-treated zebrafish showed partial podocyte foot process effacement which was more pronounced in zebrafish on nacre background compared to those on AB background (Fig. 1Cb,d).

### Influence of NaHCO_3_ concentration on zebrafish development and filtration barrier integrity

**Fig. 2.**
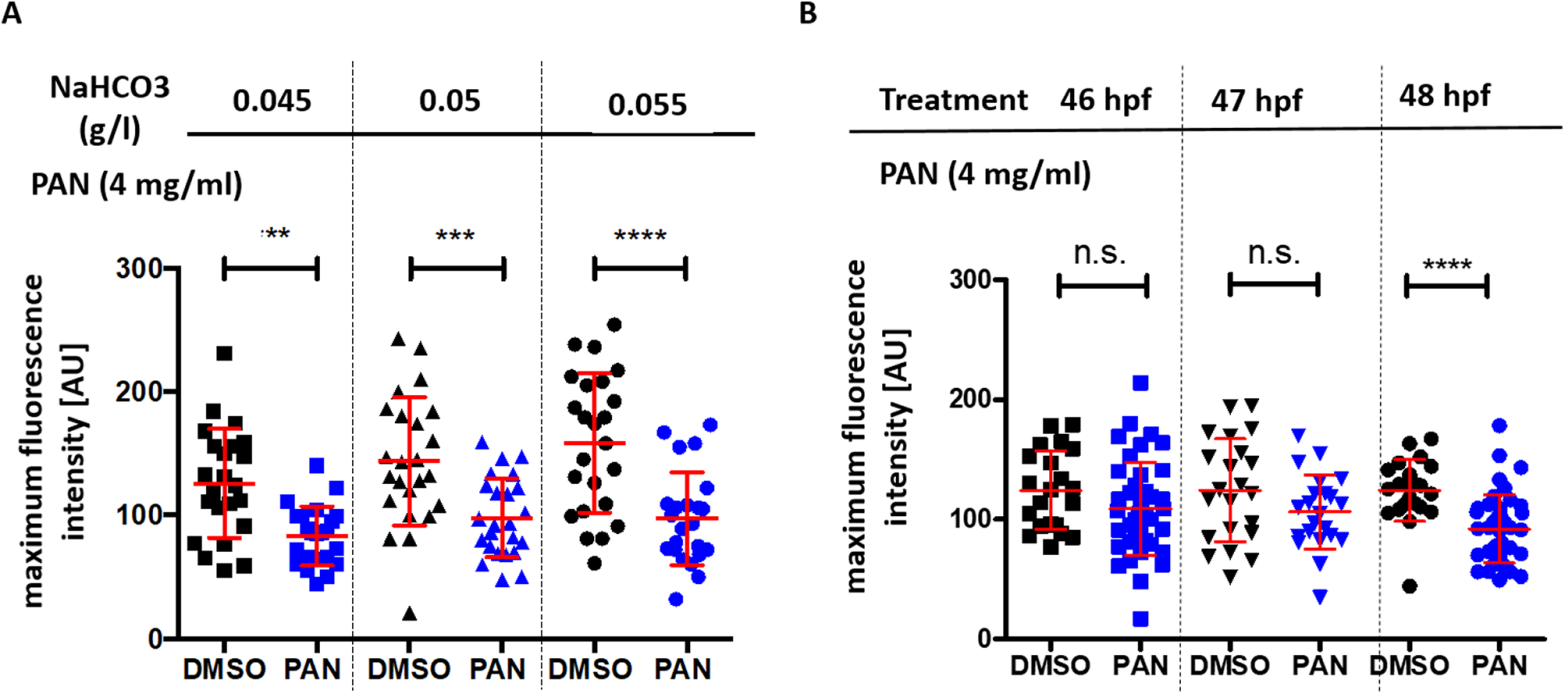
**Influence of NaHCO_3_ concentration and PAN treatment time on proteinuria.** (A) Tg(*l-fabp*:eGFP-DBP) zebrafish backcrossed of nacre (*nac^w2^*) background were treated with PAN (4 mg/ml) or DMSO at 46–48 hpf in the presence of 0.045 g/l to 0.055 g/l NaHCO_3_. Graph depicts max. fluorescence of circulating eGFP-DBP in the retinal vessel plexus of the fish eye 96 hpf. *n*=23–24 per group. (B) Tg(*l-fabp*:eGFP-DBP) zebrafish backcrossed of nacre (*nac^w2^*) background were treated with PAN (4 mg/ml) or DMSO at either 46, 47 or 48 hpf. Graph depicts max. fluorescence of circulating eGFP-DBP in the retinal vessel plexus of the fish eye 96 hpf. *n*=21–23 per group. **P<0.01, ***P<0.005, ****P<0.001, n.s., non-significant.

Additionally, because NaHCO_3_ is often used as solubilizing agent for drug delivery, we aimed to rule out that NaHCO_3_ would, on its own, have an effect on zebrafish development or glomerular filtration barrier function. For detection of proteinuria we used a transgenic fish line that expresses a green fluorescent plasma protein that can be easily quantified in the retinal vessel plexus of the zebrafish (Tg(*l-fabp*:eGFP-DBP)) (Xie et al., 2010). Loss of this green plasma protein from the circulation is suggestive of proteinuria in this model. Tg(*l-fabp:eGFP*-DBP) zebrafish with a nacre (*nac^w2^*) background were grown in different NaHCO_3_ concentrations in the embryo rearing medium and treated with DMSO only or PAN (4 mg/ml) from 46 to 48 h post fertilization (hpf). NaHCO_3_ concentrations ranging from 0.045 to 0.055 g/l had no effect on proteinuria alone and did not alter the efficacy of PAN (Fig. 2A).

### Influence of treatment time on proteinuria phenotype

Furthermore, we wanted to test the optimal time for PAN treatment for consistency. We used Tg(*l-fabp*:eGFP-DBP) zebrafish on nacre background and added PAN in a concentration of 4 mg/ml into the fish water at 46, 47 or 48 hpf. Intensity of circulating eGFP-DBP in the retinal vessel plexus of the fish eye was analyzed at 96 hpf. PAN treatment at 46 hpf yielded consistent PAN-induced phenotypes (Fig. 2B). We continued with 46 hpf as treatment time point for the following experiments.

### Dose response of PAN treatment and recovery following PAN treatment

**Fig. 3.**
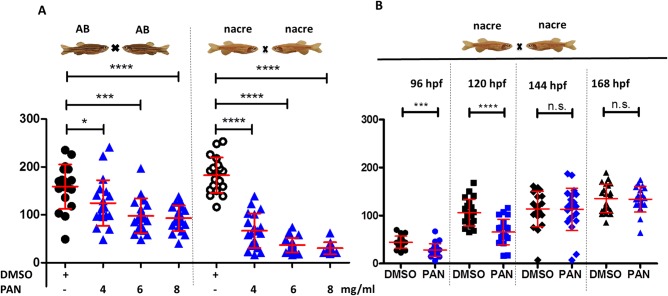
**Dose response of PAN treatment and recovery following PAN treatment.** (A) Tg(*l-fabp*:eGFP-DBP) zebrafish backcrossed on AB or nacre (*nac^w2^*) background were treated with 4 mg/ml, 6 mg/ml or 8 mg/ml PAN or DMSO at 46 hpf. Graph depicts max. fluorescence of circulating eGFP-DBP in the retinal vessel plexus of the fish eye 96 hpf. *n*=11–21 per group. (B) Tg(*l-fabp*:eGFP-DBP) zebrafish backcrossed on nacre (*nac^w2^*) background were treated with 4 mg/ml PAN at 46 hpf. Intensity of circulating eGFP-DBP (arbitrary units) in the retinal vessel plexus of the fish eye were measured at 96, 120, 144 and 168 hpf. *n*=19–21 per group. *P<0.05, ***P<0.005, ****P<0.001, n.s., non-significant.

To check if there was a correlation between increasing PAN dosage and proteinuria, zebrafish were treated with a concentration of 4 mg/ml, 6 mg/ml or 8 mg/ml PAN in the fish water. Increasing PAN concentration led to increased loss of plasma proteins in a concentration-dependent manner (Fig. 3A). For the development of a drug testing assay to identify protective or therapeutic agents for glomerular diseases, it was important to know how stable and lasting the PAN injury model is. Spontaneous recovery from injury could result in false positive interpretations on drug efficiency. To test how long the PAN effect in the zebrafish would last, Tg(*l-fabp*:eGFP-DBP) zebrafish of nacre (*nac^w2^*) background were treated with PAN in a concentration of 4 mg/ml in the fish water at 46 hpf and the fluorescent signal of the eGFP-DBP was measured in the retinal plexus of the zebrafish at 96, 120, 144 and 168 hpf. At 96 and 120 hpf, zebrafish larvae lost significant amounts of plasma proteins compared to DMSO-treated fish. However, at the later time points of 144 and 168 hpf, there was no difference in the intensity of the eGFP-DBP in the retinal vessel, indicating that fish had recovered from PAN-induced injury (Fig. 3B). Therefore, only the 96 and 120 hpf time points would be stable time points for screening assays.

### Mitf knockdown model and PAN treatment

**Fig. 4.**
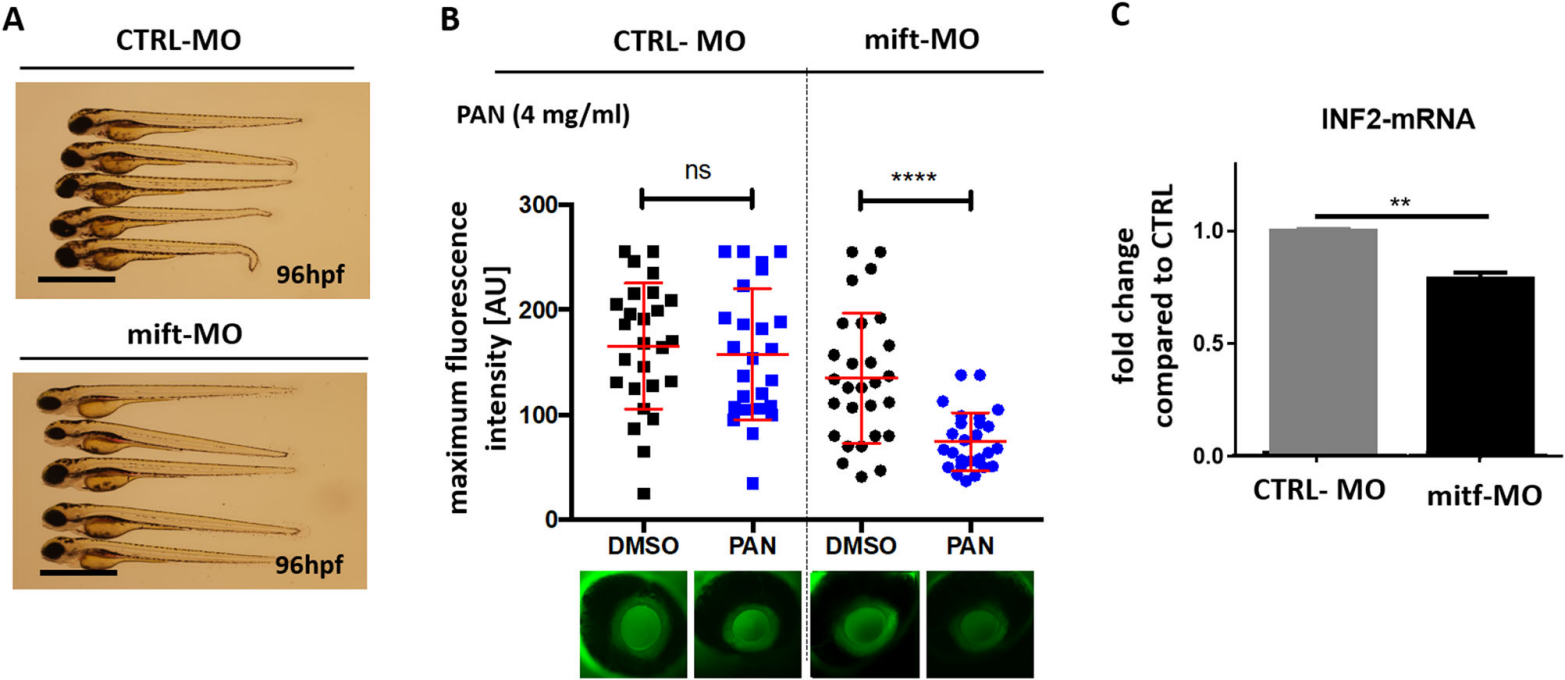
**Mitf knockdown model and PAN treatment.** (A) Phenotype pictures of Tg(*l-fabp*:eGFP-DBP) zebrafish backcrossed on AB background that were injected with either a *mitf* MO or a scrambled MO in a concentration of 250 µM in one to four-cell stage. Pictures were taken at 96 hpf. (B) Tg(*l-fabp*:eGFP-DBP) zebrafish backcrossed on AB background were injected with a mitf MO or a scrambled MO in a concentration of 250 µM in one to four-cell stage. At 46 hpf fish were treated either with PAN (4 mg/ml) or DMSO. Intensity of circulating eGFP-DBP (arbitrary units) in the retinal vessel plexus of the fish eye was measured at 96 hpf. Representative images of the fluorescence of circulating eGFP-DBP in the retinal vessel plexus of the fish eye 96 hpf are shown at the bottom. *n*=25–28 per group. (C) Q-PCR for inf2 mRNA expression in whole zebrafish at 120 hpf. Zebrafish (AB strain) were injected with CTRL-MO or a mitf-MO at one to four-cell stage. Data from three different experiments. Significance was tested with two-way ANOVA: **P<0.01, ****P<0.001, ns, non-significant. Scale bars: 500 µm.

In order to prove whether a reduction in mitf expression is the cause of the higher susceptibility of nacre fish towards PAN treatment, we injected a specific morpholino (MO) targeting mitf in Tg(*l-fabp*:eGFP-DBP) zebrafish embryos with an AB background. After 96 hpf, the mitf-MO-injected zebrafish had developed less pigment compared to CTRL-MO-injected fish (Fig. 4A). Next, mitf-MO and the CTRL-MO-injected Tg(*l-fabp*:eGFP-DBP) zebrafish of AB background were treated with PAN from 46 hpf to 96 hpf. At 96 hpf, the loss of plasma proteins from the fish was quantified. We confirmed that zebrafish with an mitf knockdown were significantly more sensitive to PAN-induced proteinuria compared to control fish (Fig. 4B). We speculate that altered formin expression in nacre zebrafish caused the increase in their susceptibility towards PAN. Interestingly, mitf-MO-injected zebrafish showed a slight but significant reduction of inf2 mRNA expression at 120 hpf compared to the inf2 mRNA expression in CTRL-MO-injected zebrafish (Fig. 4C).

### Mechanism of increased susceptibility to PAN treatment

**Fig. 5.**
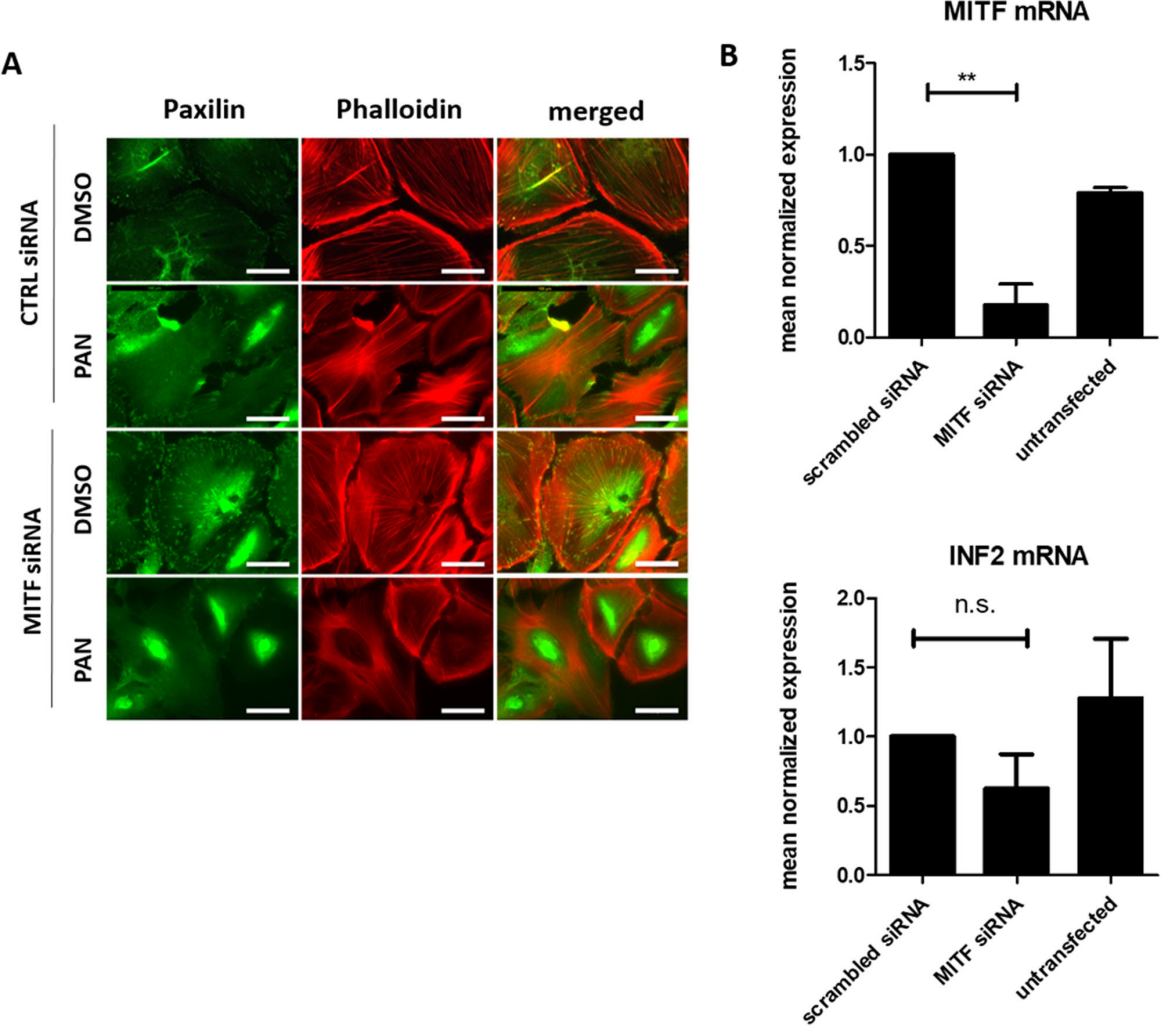
**Mechanism of increased susceptibility to PAN treatment.** (A) Immunofluorescence pictures of cultured human podocytes stained with Anti-Paxillin (green) and Phalloidin (red) to illustrate the cytoskeleton. Cells were either transfected with CTRL siRNA or a MITF siRNA and treated with DMSO or PAN 120 h after transfection. (B) Q-PCR for MITF mRNA (upper panel) and INF2 mRNA expression (lower panel) in cultured human podocytes. Cells were either transfected with CTRL siRNA, MITF siRNA or left untransfected. A reduced expression of MITF mRNA after MITF^KD^ with siRNA was present. MITF^KD^ induced a tendency to reduce INF2 mRNA expression compared to CTRL siRNA transfected cells. **P<0.01, n.s., non-significant. Scale bars: 50 µm.

To investigate the higher susceptibility towards glomerulopathy after MITF^KD^ further, we studied the effect of PAN on the actin cytoskeleton in cultured human podocytes. Differentiated human podocytes were transfected with an MITF siRNA or a control siRNA followed by treatment either with PAN or DMSO by adding the substances to the cell culture media. PAN treatment of human podocytes caused a rearrangement of the actin cytoskeleton that was more severe in podocytes that were previously transfected with the MITF siRNA (Fig. 5A). Visualization of F-actin with fluorescent phalloidin revealed that stress fibers of the actin cytoskeleton are arranged in a ring-like structure at the edge of the cells in MITF^KD^ podocytes treated with PAN. Transfection with MITF siRNA did not only decrease MITF mRNA expression, but also showed a trend to reduce INF2 mRNA expression in cultured human podocytes (Fig. 5B).

## DISCUSSION

Zebrafish larvae are widely used by different investigators as a model system to study glomerular diseases. In the past, knockdown of genes by different techniques has been used to mimic glomerular disease conditions. Here we describe an easy to use, standardized high-throughput model for glomerular damage in zebrafish via administration of PAN in fish water. The phenotypic outcome is consistent with the induction of a podocytopathy in the fish as previously described in the PAN-rat model. This method allows for subsequent testing of protective and therapeutic drugs. NaHCO_3_ concentration did not influence PAN effects, which is an important result for later drug testing as many drugs are soluble in buffers containing NaHCO_3_.

Interestingly, we found that the genetic background of the fish influenced the susceptibility of the fish to renal injury by PAN. Fish of the nacre background exhibited a higher level of proteinuria and displayed a more pronounced effacement phenotype. The nacre zebrafish line resulting from a mitf mutation was first described by Lister et al. (1999). Mitf regulates differentiation, proliferation and survival of melanocytes, hence the general lack of pigment in the nacre fish line (Hartman and Czyz, 2015). Mitf is also a transcription factor that regulates the expression of other genes. Low Mitf levels are associated with the reorganization of the actin cytoskeleton in melanomas via regulation of diaphanous-related formin Dia1 (Carreira et al., 2006). Formins are actin-binding proteins that have multiple effects on actin dynamics (Gaillard et al., 2011; Esue et al., 2008). We found a reduced inf2 mRNA expression in zebrafish from an AB strain that were injected with a mitf-MO, while an MITF^KD^ in human podocytes had a tendency to reduce INF2 mRNA expression. Since it has been previously shown that the mutations in podocyte INF2 in humans cause FSGS, INF2 associated with varying expression levels of MITF may play a role in this context (Brown et al., 2010; Barua et al., 2013).

The induction of inf2^KD^ in zebrafish caused a glomerular phenotype showing pericardial effusion and yolk sac edema (Sun et al., 2014; Schenk et al., 2017). Ultrastructural analysis of the inf2^KD^ zebrafish evidenced altered cytoplasmic protrusions associated with deformed podocyte foot processes and shape alterations of the GBM (Sun et al., 2014). We speculate that altered formin expression – in particular inf2 – in nacre zebrafish causes the increase in their susceptibility towards PAN, therefore, further studies on the pathophysiologic mechanisms need to be carried out. The proposed interactions of mitf and inf2 – in particular when either protein expression is altered – could be of great relevance in understanding the dysregulation of the actin cytoskeleton induced, for example, via PAN treatment. Stabilizing components of the actin cytoskeleton can be of great therapeutic potential, as it was previously shown by our group in other animal models and human podocytes (Schiffer et al., 2015; Müller-Deile and Schiffer, 2016; Müller-Deile et al., 2016a).

Our PAN model for induction of glomerulopathy has many advantages compared to other PAN models. By adding PAN directly into the fish water, we remove the need for cardinal vein injection of the zebrafish which is associated with stress and manipulation-caused injuries for the zebrafish. Moreover, since all zebrafish are exposed to exactly the same PAN dosage in the water, application of the drug is more homogeneous than by injection, which has an inherent variability depending on the precision of the procedure. In the future, our model can be used to perform drug screenings to find substances that might be protective or therapeutic for glomerular diseases. Furthermore, mutations causing less severe phenotype and drugs with milder renal side effects might be more readily tested in the nacre fish line that has an increased susceptibility to damage.

## MATERIALS AND METHODS

### PAN treatment

Zebrafish eggs were collected within 30 min of spawning. The chorions were removed from the embryos manually with forceps just before PAN treatment. At 46, 47 or 48 hpf zebrafish were treated with either 4 mg/ml, 6 mg/ml or 8 mg/ml PAN by adding the drug into the fish medium with 1% DMSO. Fish were kept in this PAN-containing medium until 96 hpf. DMSO, which was added to the fish water in the same concentration, served as control.

### Zebrafish proteinuria assay

Tg(*l-fabp*:DBP:EGFP) zebrafish that express a green fluorescent vitamin D binding protein fused with the enhanced green fluorescent protein (eGFP-DBP) under the control of the liver-type fatty acid binding protein (*l-fabp*) promoter were used to screen for proteinuria in zebrafish larvae. The green fluorescence can easily be seen in the retinal plexus vessels of the fish. The eGFP-DBP fusion protein has a molecular weight of approximately 78 kDa. When the glomerular filtration barrier is impaired, the fish lose plasma proteins resulting in a decrease of the fluorescence intensity (Hanke et al., 2015).

### MO injection into zebrafish embryos

CTRL-MO: CCTCCTACCTCAGTTACAATTTATA or Mitf-MO: CATGTTCAACTATGTGTTAGCTTCA (Nasevicius and Ekker, 2000) were injected into one- to four-cell stage zebrafish embryos using a Nanoject II injection device (Drummond Scientific, Broomall, PA). MOs were ordered from GeneTools (Philomath, OR,). Injections were carried out in injection buffer (100 mM KCl, 0.1% phenol red). The animal protocol was approved by the MDI Biological Laboratory IACUC #17-03 and is in line with the MDIBL international assurance #D16-00341.

### Transmission electron microscopy of zebraﬁsh glomeruli

Zebraﬁsh larvae were ﬁxed at 120 hpf in solution D and embedded EPON (recipe/protocol from EMS, Hatﬁeld, PA). Semithin (300 nm) and ultrathin (90 nm) sectioning was performed with a Leica Microtome (Leica Microsystems Inc., Buffalo Grove, IL) and transferred onto copper slit grids (Electron Microscopy Sciences, Hatﬁeld, PA). Grids were stained with uranyl acetate (2%) for 30 min and lead citrate for 15 min with three washing steps in between.

### Transfection of human podocytes with siRNA and treatment with PAN

Human podocytes were cultured and differentiated in RPMI medium (10% FCS, 1% Penicillin/Streptomycin, 0.1% human insulin). 10,000 cells per well were seeded onto 24-well plates and differentiated for 7 days. An MITF siRNA and a control siRNA were transfected into the cells in a concentration of 10 nM using transfection reagent and buffers from OriGene (siTRAN 1.0). 4 h after transfection, medium was changed to fresh RPMI medium. 120 h after transfection cells were treated with 50 µg/µl PAN for 48 h.

### Immunofluorescence

Human podocytes were fixed with 4% PFA at room temperature for 10 min and washed with PBS. 0.1% Triton for 10 min was used to permeabilize the cells. 10% donkey serum for 30 min was used to block unspecific binding of the antibody. Alexa Fluor 594-labeled Phalloidin (Invitrogen) and Anti-Paxilin antibody (Millipore) were used to visualize the actin cytoskeleton.

### PCR of cultured human podocytes and of whole zebrafish

Purification of total RNA from cells was done with miRNeasy Kit (Qiagen) according to the manufacturer's protocol as shown previously after transfection of cultured human podocytes with either control siRNA or MITF siRNA. Reverse transcription was done with 1 µg RNA, Oligo(dT)primer (Promega, Madison, WI, USA), and Random primer (Promega) that were incubated at 70°C for 10 min followed by an incubation with M-MLV RT buffer (Promega), dNTPs (Roche, Mannheim, Germany), and M-MLV reverse transcriptase (Promega) at 42°C for 90 min and at 70°C for 10 min. For mRNA targets, we used sybr green-based real-time PCR with the following protocol: 1 min at 95°C followed by 35 cycles of 10 s at 95°C, 10 s at 60°C and 10 s at 72°C followed by 5 s at 95°C and 1 min at 65°C.

Sequences of human MITF primers were: forward primer 5′→3′ TACCACATACAGCAAGCCCA; reverse primer 5′→3′ACGCTCGTGAATGTGTGTTC.

Sequences of human INF2 primers were: forward primer 5′→3′GCCAAGAAGAGCCTGAACCT; reverse primer 5′→3′TCAATCTCGTGCTTCTCGG.

Sequences for zebrafish inf2 primers were: forward primer 5′→3′ TGGCATTCACTTCATCGTGGA; reverse primer 5′→3′ TGGACGTATCCAAAGCTTGC.

Sequences of human HPRT primers were: forward primer 5′→3′CAGTCCCAGCGTCGTGATTA; reverse primer 5′→3′ AGCAAGTCTTTCAGTCCTGTC3.
